# VviAMT4;1 Is a High-Affinity Ammonium Transporter in Table Grape

**DOI:** 10.3390/plants15030519

**Published:** 2026-02-06

**Authors:** Huilin Xiao, Matthew Shi, Yanwen Tang, Rui Yuan, Zhizhong Song, Meiling Tang

**Affiliations:** 1 Yantai Academy of Agricultural Sciences, Yantai 264000, China; huilinx0001@163.com; 2 College of Horticulture, Ludong University, No. 186 Hongqizhong Road, Yantai 264025, China; ss2619@cam.ac.uk (M.S.); 18815705840@163.com (Y.T.); yuanruiwh@163.com (R.Y.); 3 Wolfson College, University of Cambridge, Cambridge CB3 9BB, UK; 4 Department of Plant Science, University of Cambridge, Cambridge CB2 3EA, UK

**Keywords:** table grape, AMT2 transporters, NH_3_ transport, functional complementation in yeast mutant, substrate feedback inhibition

## Abstract

Ammonium transporters (AMTs) are a class of membrane-associated proteins that play crucial roles in the uptake and transport of ammonium (NH_4_^+^ or NH_3_). In this study, an ammonium transporter-encoding gene *VviAMT4;1* was isolated and identified from table grape ‘Yanpu No.2’. Notably, the expression level of *VviAMT4;1* varied significantly across different organs or tissues of ‘Yanpu No.2’, and the highest expression level was detected in the roots of both tissue-cultured seedlings and 5-year-old mature trees. Expression of *VviAMT4;1* was significantly up-regulated under NH_4_^+^ depletion throughout the whole of tissue-cultured seedlings. Yeast mutant functional complementation indicates that the recombinant strain pYES2-VviAMT4;1/31019b restored growth under different pH conditions. ^15^N isotope-labeled uptake kinetics analysis demonstrated that VviAMT4;1 is a typical high-affinity ammonium transporter, with a *Kₘ* value of 49.58 ± 4.66 μmol·L^−1^ and a *Vₘₐₓ* value of 3.29 μmoles·min^−1^·μg^−1^ cells. Moreover, VviAMT4;1 can mediate the weak uptake and utilization of methyl amine (MeA^+^) in yeast cells. The VviAMT4;1-mediated NH_4_^+^ uptake process may suffer from feedback inhibition by endogenous NH_4_^+^ enrichment. This study provides insights into understanding the molecular mechanisms of N transport and utilization in fruit trees.

## 1. Introduction

Ammonium serves as a primary nitrogen source for plants and exhibits significant absorption advantages under low-nitrogen conditions (soil NH_4_^+^ concentration < 1 mmol·L^−1^), with its transmembrane transport efficiency exceeding that of nitrate by 3–5 times. However, when the intracellular ammonium concentration exceeds a critical threshold (typically >5 mmol·L^−1^), it triggers harmful effects such as proton imbalance and membrane potential disorder [1,2]. The precise regulation of this “dual effect” relies on the functional synergy of the ammonium transporter (AMT) family [3,4,5].

In plants, AMT transporters are mainly located on the plasma membrane, containing 10 or 11 typical conserved transmembrane regions, and transport ammonium via a high- or low-affinity transport system [3,4,5,6]. Plant AMTs are divided into two major sub-groups, AMT1 and AMT2, which share similar topological structures and mediate ammonium absorption and transport functions in the form of trimers [3,5]. Since the cloning of *AtAMT1;1* in *Arabidopsis thaliana* [7], dozens of AMT1 subgroup homologous genes have been identified in annual plants such as tomato (*Solanum lycopersicum*) [8,9], rice (*Oryza sativa*) [10,11,12], alligatorweed (*Alternanthera philoxeroides*) [13], and wheat (*Triticum aestivum*) [14].

Common methods for functional research include T-DNA insertion gene mutation, electrophysiological technology, and yeast heterologous expression systems [3,9,11,12,13,14,15]. Research shows that *OsAMT1;1* in rice exhibits a constitutive expression pattern, with expression in both the aerial parts and roots. In contrast, *OsAMT1;3* displays root-specific expression characteristics. The expression of both *OsAMT1;1* and *OsAMT1;3* is induced by nitrogen starvation but repressed via glutamine-mediated feedback [10]. Using electrophysiological technology and complementation of functional yeast mutant, Yang et al. [11] verified that OsAMT1;1 is an ammonium transporter with relatively low affinity for ammonium, with a affinity constant (*Km*) of 110–129 μmol·L^−1^. Hao et al. [12] demonstrated that OsAMT1;3 is a typical high-affinity ammonium transporter, with a *Km* of approximately 32 μmol·L^−1^ and weak permeability to methylammonium (MeA^+^).

The AMT transporters play a key role in regulating ammonium uptake and maintaining nitrogen homeostasis in plants. Research on the agronomic traits of plant AMT transporters is primarily focused on the AMT1 subgroup, while studies on AMT2 agronomic traits and their regulation of ammonium uptake and metabolism remain relatively scarce. Studies using *Arabidopsis* T-DNA insertion or transposon mutants indicate that AtAMT1;1 and AtAMT1;3 play a dominant role in high-affinity ammonium absorption. The AtAMT1;1 single mutant results in a 30% reduction in high-affinity ammonium absorption in plants [16], while the transposon insertion mutant of AtAMT1;2 leads to a 28% reduction in high-affinity ammonium absorption in roots. The double mutant of AtAMT1;1 and AtAMT1;3 significantly inhibits plant growth, with a 70% decline in ammonium absorption capacity [15,17]. Nonetheless, AtAMT1.1, AtAMT1.2, and AtAMT1.3 together contribute approximately 95% of ammonium uptake. Homologs of these AMT proteins in rice have been reported to mediate ammonium uptake in roots and are highly expressed under conditions of excess ammonium supply [18].

Currently, plant AMT2 transporters are characterized as NH_3_ transporters [19,20]. In *Arabidopsis*, AtAMT2 functions as a channel-like NH_3_ flux transporter [19]. In maize, ZmAMT3;1 mediated high-affinity ammonium transport, with the substrate NH_4_^+^ being accessed, but actually transporting NH_3_ [20]. However, the biological functions of AMT2 transporters in fruit trees are essentially unexplored. In peach, *PpeAMT3;4* is primarily expressed in roots and significantly down-regulated by ammonium toxicity. A ^15^N-labeled ammonium uptake assay in yeast cells revealed that *PpeAMT3;4* is a typical high-affinity ammonium transporter [6]. In pear, *PbAMT2* is expressed in all tested organs with the highest level detected in roots, whereas *PbAMT3* is primarily expressed in leaves [21].

As a globally important perennial vine crop, grape (*Vitis vinifera* L.) exhibits significant cultivar-specific characteristics in nitrogen metabolism. Fruit quality parameters (e.g., soluble solids, anthocyanin content) show a strong positive correlation with nitrogen supply, and over 60% of flavor precursor compounds in wine are regulated by nitrogen nutrition [22,23]. In this study, *VviAMT4;1* was isolated from ‘Yanpu No.2’ and proved to be a typical high-affinity AMT2 subgroup ammonium transporter. Favorably, this work provides a theoretical foundation for elucidating the biological functions of AMT2 transporters in fruit trees.

## 2. Materials and Methods

### 2.1. Plant Material and Growth Conditions

The table grape cultivar ‘Yanpu No.2’ was used as experimental material. The tested vines were 5-year-old mature ‘Yanpu No.2’ trees provided by Yantai Academy of Agricultural Sciences, Shandong Province, and were cultivated under consistent management conditions.

Referring to the grape developmental stage classification methods [24,25], fruit samples were collected at the young fruit stage (35 days post-anthesis, DPA) and maturity (120 DPA). Flowers at the inflorescence visible stage and full bloom, as well as new leaves (fully expanded leaves at the 4th–6th internodes of new shoots) and roots (new white absorbing roots, diameter < 2 mm), were collected at different fruit developmental stages. For each sample, 3 biological replicates were selected, with 4 clusters randomly harvested from each plant. For each cluster, 30 fruits of uniform maturity, free from mechanical damage or disease, were randomly selected.

Tissue-cultured ‘Yanpu No.2’ seedlings were germinated on half-strength MS solid medium (pH 5.8) for 1 month before being transferred to half-strength MS liquid solution in plastic incubators within a growth chamber [26,27]. For ammonium depletion (–NH_4_^+^) treatment, NH_4_Cl was omitted from the MS medium. The nutrient solution was changed every other day.

All collected samples were immediately frozen in liquid nitrogen and stored at −80 °C for subsequent analysis.

### 2.2. Isolation and Cloning of VviAMT4;1

Using the coding protein sequences of the peach PpeAMT3;4 and PpeAMT4;1 genes as the reference sequences [6], a potential grape AMT gene was retrieved from the Phytozome grape genome database and named *VviAMT4;1*. The following primers were designed: forward primer 5′-ATGGCGTCCCTGGACTGCTC-3′ (20 bp sequence starting from the initiation codon) and reverse primer 5′-TTAACAATGACGGCACATGG-3′ (20 bp sequence preceding the termination codon). Total RNA was extracted from the roots of ‘Yanpu No.2’ tissue-cultured seedlings, and first-strand cDNA was synthesized using the PrimeScript™ RT Reagent Kit (TaKaRa, Dalian, China) as the template. The *VviAMT4;1* gene was amplified using PrimeSTAR™ HS DNA Polymerase (TaKaRa, Dalian, China) and sent to Sangon Biotech (Shanghai, China) Co., Ltd. for sequencing verification.

### 2.3. Phylogenetic Tree Construction of Plant AMT Homologues

Amino acid sequence alignment of AMT homologous proteins, including table grape VviAMT4;1, *Prunus persica* PpeAMT3;4 and PpeAMT4;1, *Lotus japonicus* LjAMT2;1, *Populus trichocarpa* PtrAMT4;1, *Oryza sativa* OsAMT2;1 and OsAMT4;1, and *Arabidopsis thaliana* AtAMT2;1, was conducted using ClustalX_2.0.13 software. Amino acid sequences of AMT homologues were collected from table grape, *S. lycopersicum*, *P. persica*, *L. japonicus*, *P. trichocarpa*, *O. sativa*, and *A. thaliana*. A phylogenetic tree of plant AMT homologues was constructed using the maximum likelihood method in MEGA 15.0, with a bootstrap test performed using 1000 replicates to assess the confidence of the tree.

### 2.4. Quantitative Real Time PCR (qRT-PCR)

Using the online NCBI/Primer-BLAST server, sequence-specific primers for *VviAMT4;1* were designed (forward primer: 5′-CACCGTATGGACCAGGCTTAT-3′; reverse primer: 5′-GGCATACGCTTCCTCTCCAT-3′). Grape *Ubiquitin* (GenBank No. MH114011) and *Tubulin* (GenBank gene ID: 100246164) were used as internal reference genes [24,25,26,27,28,29,30]. The expression patterns of *VviAMT4;1* in different tissues at various developmental stages were analyzed using SYBR Green (TaKaRa, Dalian, China) on an ABI 7500 Real-Time PCR System (ABI, New York, NY, USA). The PCR reaction was performed under the following conditions: 95 °C for 2 min, 1 cycle; 95 °C for 30 s, 60 °C for 30 s and 72 °C for 30 s, 28 cycles; 72 °C for 10 min, 1 cycle. Each reaction was performed with four biological replicates. The *Ct* values obtained from the ABI 7500 PCR system were normalized using the internal reference genes, and relative expression levels were calculated using the 2^−ΔΔCt^ method [6,13,24,25,26,27,28,29,30].

### 2.5. Functional Complemention of VviAMT4;1 in Yeast Mutant

The recombinant plasmid pYES2-*AMT4;1* was constructed by cloning the CDS of the *VviAMT4;1* gene into the pYES2 vector [11,12,13,26]. This process utilized the forward primer 5′-GAGAGGTACCATGGCGTCCCTGGACTGCTC-3′ (*Kpn* I underlined) and reverse primer 5′- GAGAGTCGACTTAACAATGACGGCACATGG -3′ (*Sal* I underlined). The resulting recombinant plasmid was subcloned into the triple-mutant yeast strain 31019b (*MATa mepl△ mep2△::LEU2 mep3△::KanMX2 ura3*) [11,12,13,26]. The empty vector pYES2 and the recombinant vector pYES2-AMT4;1 were separately transformed into 31019b via electroporation. Then, 1 mL of pre-chilled 1 mol·L^−1^ sorbitol solution was immediately added, and the cells were allowed to recover at 30 °C for 2 h before being plated onto yeast selection medium (0.17% YNB + 2 mmol·L^−1^ arginine + 2% D-galactose + 2% agar, pH 5.8). The plates were incubated at 30 °C in the dark for 72 h. Single colonies were picked and inoculated into liquid selection medium, followed by incubation at 30 °C with shaking at 200 rpm for 36 h. Positive recombinant yeast clones were confirmed by PCR. The positive strains were cultured in YNB liquid selection medium until the OD_600_ reached 1.0. Tenfold serial dilutions (10^0^, 10^−1^, 10^−2^, 10^−3^) were prepared, and 5 μL of each dilution was spotted onto solid test plates containing either 2 mmol·L^−1^ arginine or 2 mmol·L^−1^ NH_4_Cl as the sole nitrogen source (other components were the same as the screening medium).

To examine the effects of methyl amine (MeA^+^) or L-methionine sulfoximine (L-MSX) on the ammonium uptake activity of *VviAMT4;1*, 3 mmol·L^−1^ MeA^+^ or 20 μmol·L^−1^ L-MSX was added to the solid medium with 2 mmol·L^−1^ NH_4_Cl as the sole nitrogen source [11,12,13]. The plates were incubated at 30 °C in the dark for 72 h, and colony growth was recorded using a gel imaging system. Parallel control experiments were performed with the empty vector-transformed strain (pYES2/31019b).

### 2.6. Kinetic Analysis of ^15^N Isotope Tracer-Labeled Ammonium Uptake

Following the methods previously reported by Yang et al. [11] and Guo et al. [13], the ammonium uptake kinetics of VviAMT4;1 were analyzed. Yeast cells were cultured in YNB liquid medium containing ^15^N isotope-labeled ^15^NH_4_Cl, with NH_4_^+^ concentrations set at 0, 0.01, 0.025, 0.05, 0.1, 0.25, 0.5, 1, and 2 mmol·L^−1^. The ^15^N-labeled ^15^NH_4_^+^ content was measured using a FLASH EA-DELTA V Elemental Analyzer-Mass Spectrometer (Flash-2000 Delta V ADVADTAGE, Thermo Fisher Scientific, Norristown, PA, USA), and the kinetic parameters *Km* (affinity constant) and *Vmax* (maximum uptake rate) were derived from curve fitting [11,12,13].

### 2.7. Statistical Analysis

Graphs were generated using Origin 12.0 software, and significant differences were analyzed using Student’s *t*-test in SPSS 13.0 software (SPSS Chicago, IL, USA), with details provided in the legends.

## 3. Results

### 3.1. Isolation of VviAMT4;1 from Table Grape

**Figure 1:**
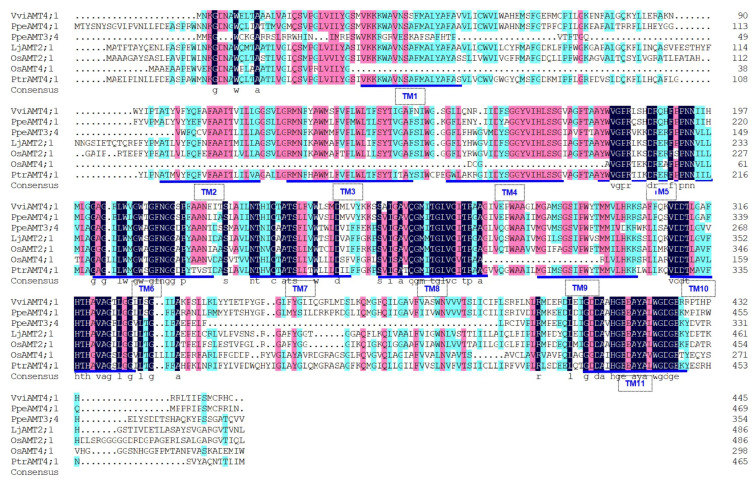
Amino acid alignment of plant AMT homologues. AMT proteins were chosen from table grape VviAMT4;1, *P*. *persica* PpeAMT3;4 and PpeAMT4;1, *L*. *japonicus* LjAMT2;1, *P*. *trichocarpa* PtrAMT4;1, *O*. *sativa* OsAMT2;1 and OsAMT4;1, and *A*. *thaliana* AtAMT2;1. The colors of black, pink, and dark green indicate identities of 100%, 85%, and the range between 45% and 70%, respectively, at the same amino acid residue. Note: TM means transmembrane region, underlined by blue lines.

Using the peach AMT3;4 protein sequence as a reference [6], a homologous protein (VIT_200s1818g00010) was identified in the Phytozome grape genome, which was named *VviAMT4;1*. InterProScan prediction revealed the presence of an ammonium transporter functional domain, suggesting it might be a potential ammonium transporter protein. The *VviAMT4;1* gene was further amplified from ‘Yanpu No.2’ roots, with a coding sequence (CDS) of 1338 bp encoding 445 amino acids (Figure 1). Gene structure analysis indicated that VviAMT4;1 has two introns, with lengths of 168 bp and 95 bp, respectively. Amino acid sequence alignment showed 64.39% identity between VviAMT4;1 and plant AMT homologues from *P. persica*, *L. japonicus*, *P. trichocarpa*, *O. sativa*, and *A. thaliana*. In addition, these plant AMT homologues contain 11 typical transmembrane domains (Figure 1).

### 3.2. Phylogenetic Tree Construction and Analysis

**Figure 2:**
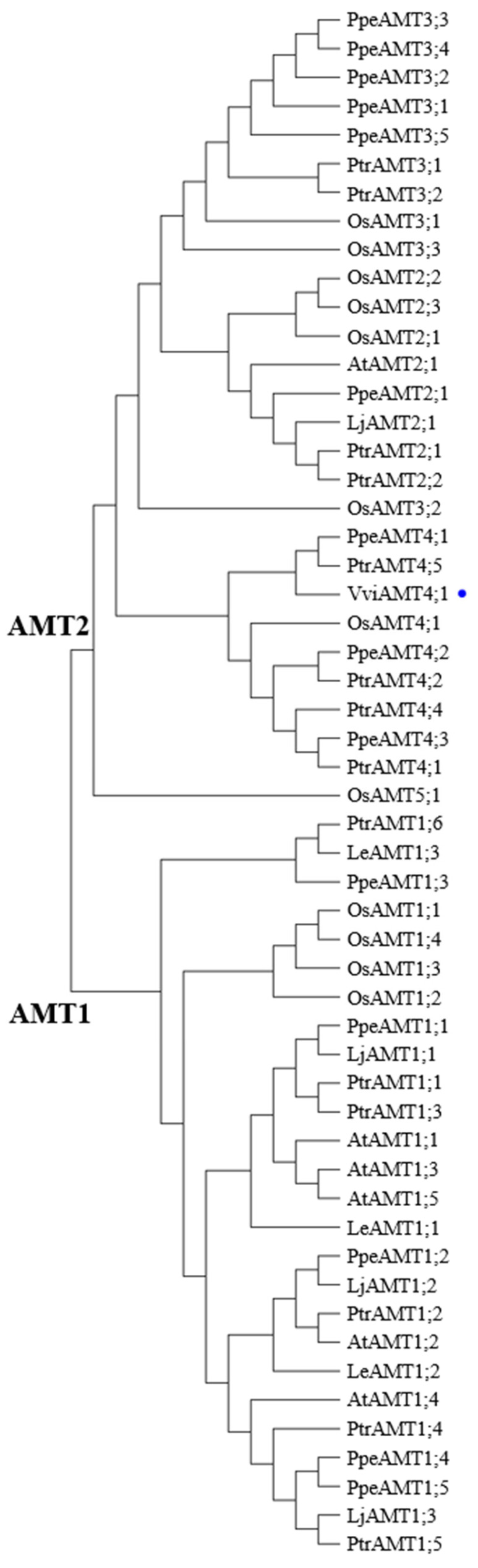
Phylogenetic tree of plant AMT homologues. The tree was constructed using the maximum likelihood method in MEGA 15.0. Amino acid sequences of AMT homologues were collected from table grape, *S*. *lycopersicum*, *P*. *persica*, *L*. *japonicus*, *P*. *trichocarpa*, *O*. *sativa*, and *A*. *thaliana*. Table grape VviAMT4;1 is labelled with a blue dot.

Phylogenetic tree analysis revealed that plant AMT transporters are classified into two major subgroups: AMT1 and AMT2. Notably, VviAMT4;1 was tightly clustered with peach PpeAMT4;1 and poplar PtrANT4;5, which belong to the AMT2 subgroup. The clustering pattern suggests that AMT2 subgroup transporters, particularly those of perennial tree species, share a relatively close evolutionary distance (Figure 2).

### 3.3. Expression Profiles of VviAMT4;1

**Figure 3:**
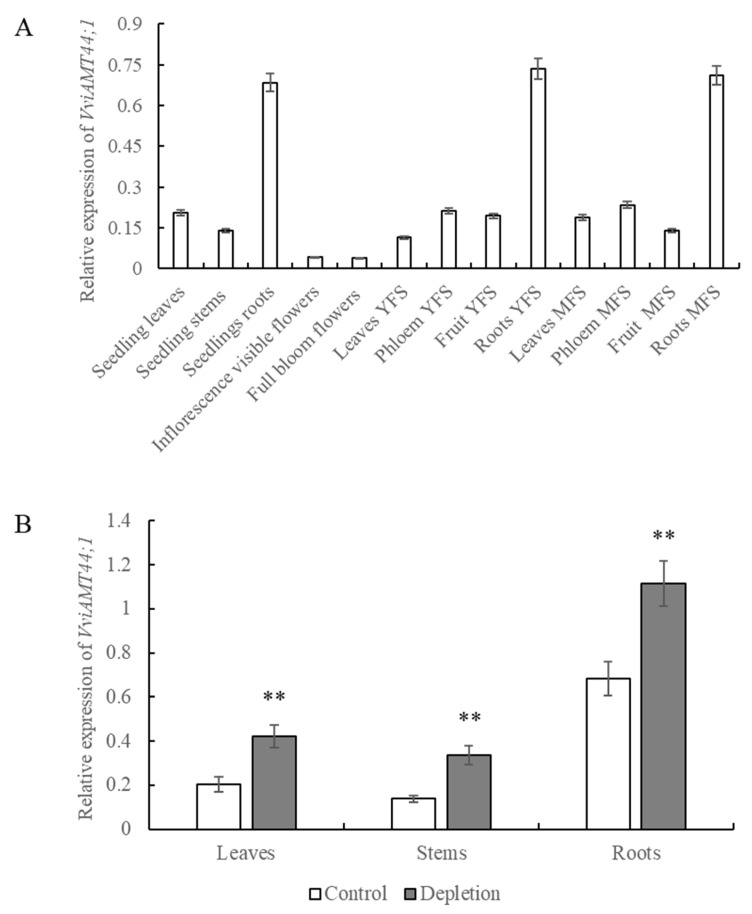
Tissue-specific expression analysis of *VviAMT4;1*. (**A**) Relative expression of *VviAMT4;1* in 5-year-old mature ‘Yanpu No.2’ trees. (**B**) Relative expression of *VviAMT4;1* in tissue-cultured ‘Yanpu No.2’ seedlings. Tissue samples were collected and frozen immediately in liquid nitrogen before qRT-PCR analysis. YFS, young fruit state; MFS, mature fruit stage. Asterisks indicate statistical difference found between the control and NH_4_^+^ depletion (** *p* < 0.01).

Both grape *Ubiquitin* (GenBank No. MH114011) and *Tubulin* (GenBank gene ID: 100246164) were evaluated as internal reference genes, and similar expression trends were obtained. In this study, results are presented using *Ubiquitin* as the internal reference gene (Figure 3). Tissue-specific expression analysis revealed that the *VviAMT4;1* gene was most highly expressed in the roots of both mature ‘Yanpu No.2’ grapevines and tissue-cultured seedlings. In contrast, its expression levels were relatively low in other tissues or organs, including leaves and stems of seedlings, as well as flowers, leaves, phloem, and fruits of mature vines (Figure 3A).

Moreover, expression of *VviAMT4;1* was strongly induced by NH_4_^+^ depletion. After 48 h of NH_4_^+^ starvation, the transcript levels of *VviAMT4;1* increased in all tested tissues or organs (leaves, stems, and roots) of the tissue-cultured seedlings (Figure 3B).

### 3.4. Functional Determination of VviAMT4;1 in Yeast Mutant

**Figure 4:**
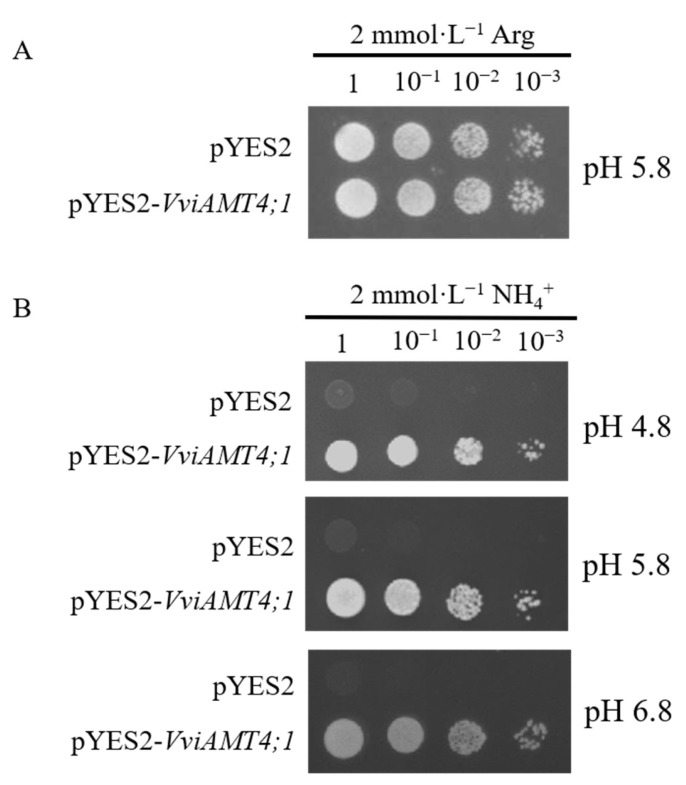
Yeast functional complementation test of *VviAMT4;1.* Yeast strain 31019b was transformed with pYES2 or pYES2-*VviAMT4;1*. (**A**) Growth assays of yeast 31019b on YNB medium supplemented with different concentrations of NH_4_Cl. Yeast cells grown on YNB medium supplemented with 2 mM arginine (Arg, pH 5.8) as the sole N source were used as positive control. (**B**) Growth assays of yeast 31019b on YNB medium under different pH conditions (pH 4.8, 5.8, and 6.8), supplemented with different concentrations of NH_4_Cl. Final diluted concentrations are indicated by 1, 10^−1^, 10^−2^, and 10^−3^, respectively.

The yeast strain 31019b failed to grow normally in a medium containing less than 5 mmol·L^−1^ NH_4_^+^ as the sole nitrogen source [11,12,13]. On yeast-selective medium with 2 mmol·L^−1^ arginine (Arg) as the sole nitrogen source (pH 5.8), both the recombinant strain (pYES2-VviAMT4;1/31019b) and the empty vector-transformed strain (pYES2/31019b) exhibited normal growth. However, on the screening medium containing 2 mmol·L^−1^ NH_4_Cl as the sole nitrogen source, the empty vector-transformed strain pYES2/31019b could not grow, whereas the recombinant strain pYES2-VviAMT4;1/31019b restored growth under varying pH conditions (Figure 4). These findings suggest that VviAMT4;1 can mediate ammonium uptake in yeast cells. Meanwhile, we speculate that VviAMT4;1 may recognize NH_4_^+^ but could be implicated in NH_3_ transport.

**Figure 5:**
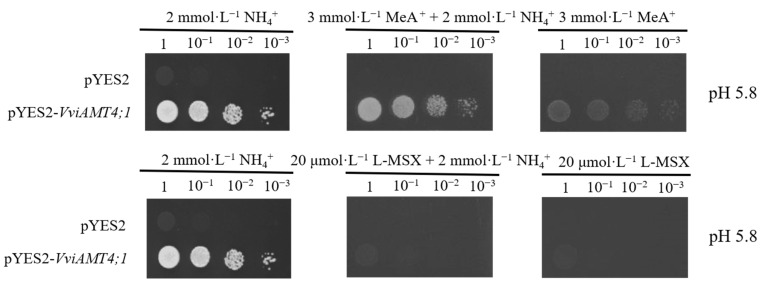
Feedback regulation analyses of VviAMT4;1-mediated ammonium uptake. Yeast cells were grown on YNB medium (pH 5.8) containing 3 mmol·L^−1^ MeA^+^ or 20 μmol·L^−1^ L-MSX as the sole N source, supplemented with 0 or 2 mmol·L^−1^ NH_4_Cl. Final diluted concentrations are indicated by 1, 10^−1^, 10^−2^, and 10^−3^.

MeA^+^ is an analog of NH_4_^+^ and significantly affects NH_4_^+^ uptake [11,12,13]. On the screening medium supplemented with both 3 mmol·L^−1^ MeA^+^ and 2 mmol·L^−1^ NH_4_Cl, the empty vector-transformed strain (pYES2/31019b) failed to grow, whereas the recombinant vector-transformed strain (pYES2-*VviAMT4;1*/31019b was able to grow but exhibited weakened growth compared to the control screening medium containing 2 mmol·L^−1^ NH_4_Cl as the sole nitrogen source. On yeast screening medium containing 3 mmol·L^−1^ MeA^+^ as the sole nitrogen source, the empty vector-transformed strain pYES2/31019b failed to grow, whereas the recombinant strain pYES2-*VviAMT4;1*/31019b exhibited weak growth (Figure 5). L-MSX is a glutamine analog that inhibits NH_4_^+^ assimilation [12,13]. On yeast screening medium containing 2 mmol·L^−1^ NH_4_Cl plus 20 μmol·L^−1^ L-MSX, or containing 20 μmol·L^−1^ L-MSX as the sole nitrogen source, neither the recombinant strain pYES2-*VviAMT4;1*/31019b nor the empty vector-transformed strain pYES2/31019b could grow (Figure 5).

### 3.5. ^15^N-Labeled Ammonium Uptake Kinetics Analysis

**Figure 6:**
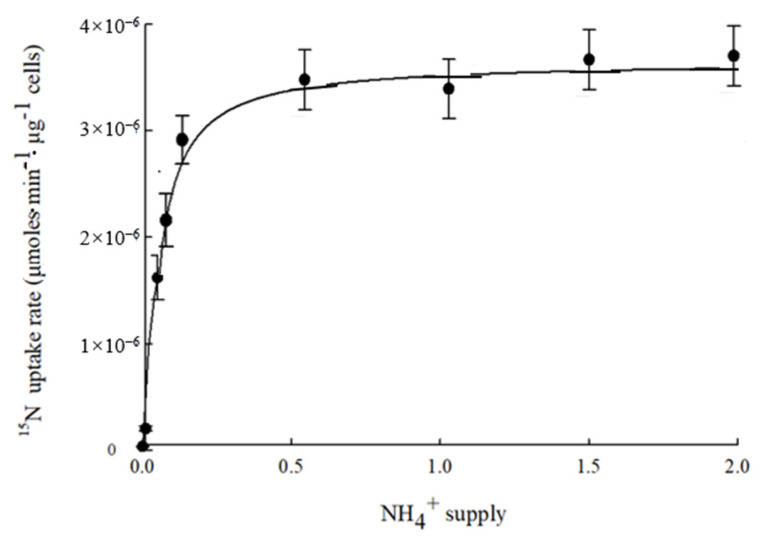
^15^N-labeled ammonium uptake analyses of VviAMT4;1 in yeast. Yeast cells transformed with pYES2 or pYES2-*VviAMT4;1* were grown in YNB liquid medium supplemented with different concentrations of ^15^N labeled ^15^NH_4_Cl. Values are presented as means ± SE, *n* = 6. Error bars within the plot symbols are not visible.

*K*m reflects the affinity between the AMT transporter and its substrate and *V*max reflects the catalytic efficiency of AMT transporters [11,12,13]. The ^15^N isotope tracer analysis revealed that VviAMT4;1-mediated ammonium uptake exhibited a *K*m of 49.58 ± 4.66 μmol·L^−1^ and a *V*max of 3.29 μmoles·min^−1^·μg^−1^ cells (Figure 6), indicating that VviAMT4;1 is a typical high-affinity ammonium transporter. 

## 4. Discussion

Ammonium is one of the primary forms of nitrogen utilized by plants. When soil nitrogen is deficient, ammonium becomes the preferred absorption form for most plant species [1,2,3,5]. In particular, AMT transporters mediate the uptake and intracellular transport of ammonium (NH_4_^+^ or NH_3_), playing a critical role in plant growth and development. The AMT family comprises over a dozen members divided into two major subgroups, AMT1 and AMT2, with the majority of their gene functions remaining uncharacterized. Current research primarily focuses on functional analyses of the AMT1 subgroup in annual model plants, like *Arabidopsis* and rice [3,4,5,6,7,8,9,10,11,12]. Due to their perennial growth habit and specific lignified stems, woody and liana plants exhibit distinct nitrogen absorption and transport mechanisms compared to herbaceous species, suggesting differences in the physiological functions and regulatory mechanisms of their AMT homologs [31,32]. Nevertheless, the biological functions of AMT2 transporters in perennial woody and liana fruit crops remain largely unclear.

In model plants, multiple AMT1 subgroup homologues, such as *Arabidopsis* AtAMT1;3 [33], rice OsAMT1;1 [10,11], OsAMT1;3 [10,12], and *Alternanthera philoxeroides* ApAMT1;1 [13], are predominantly highly expressed in roots, mediating ammonium uptake or transport in root cells [10,11,12,32]. Interestingly, an AMT2 subgroup-encoding gene, *VviAMT4;1*, was cloned from ‘Yanpu No.2’. This gene was highly expressed in roots of both tissue-cultured seedlings and 5-year-old mature trees, suggesting its potential role in ammonium uptake and transport in cells of table grape roots. Similarly, *PpeAMT4;3* was also highly expressed in peach roots [6] and *PbAMT2* was highly expressed in pear roots [21]. Simultaneously, VviAMT4;1 shares the closest evolutionary relationship with peach PpeAMT4;1 and poplar PtrAMT4;5, implying that AMT2 subgroup transporters of perennial trees may possess similar biological functions, which requires further validation. Furthermore, *VviAMT4;1* exhibits lower expression in leaves, stems, and fruits, indicating its comprehensive involvement in NH_4_^+^ allocation and transport in above-ground tissues or organs.

In plants, AMT2 family members are functionally recognized as NH_3_ transporters [19,20]. In this study, yeast functional complementation and ^15^N isotope tracer kinetic assays revealed that VviAMT4;1 is a high-affinity ammonium transporter that may be involved in NH_3_ transport, which needs further biological verification. These findings are in accordance with *Arabidopsis* AtAMT2 [19] and maize ZmAMT3;1 [20]. Together, these findings support the proposition that NH_4_^+^ is firstly recruited by the binding site and then transported across the membrane in the form of uncharged NH_3_ by dehydrogenation in AMT2 transporters.

Notably, MeA^+^ impacts ammonium uptake [11,12,13]. This study demonstrates that MeA^+^ as a substrate analog inhibits ammonium uptake and utilization in yeast cells, consistent with findings in rice OsAMT1;1 [11] and OsAMT1;3 [12] and *A. philoxeroides* ApAMT1;1 [13]. These findings suggest that VviAMT4;1 may play a role in nitrogen nutrition dynamics and efficient utilization in table grape roots. In addition, when ammonium and MeA^+^ coexist, VviAMT4;1 mediates preferential ammonium utilization, ensuring weak growth of yeast mutants. However, when MeA^+^ is the sole nitrogen source, VviAMT4;1 can also facilitate trace MeA^+^ uptake. The addition of L-MSX abolished VviAMT4;1-mediated ammonium uptake, aligning with results from rice OsAMT1;3 [12] and alligatorweed ApAMT1;1 [13]. Since L-MSX is a glutamine analog primarily inhibiting glutamine synthetase [12,13], it is speculated that ammonium absorbed by plant AMTs may participate in glutamine synthesis. L-MSX blocks this pathway, limiting ammonium assimilation and thereby suppressing VviAMT4;1 function. These findings indicate that VviAMT4;1 may be subject to feedback inhibition by intracellular ammonium accumulation.

Favorably, this study provides genetic resources for elucidating the biological functions of AMT2 transporters in table grapes and lays a theoretical foundation for exploring the molecular mechanisms of VviAMT4;1 in efficient nitrogen utilization.

## 5. Conclusions

*VviAMT4;1* was isolated from table grape ‘Yanpu No.2’. It was highly expressed in roots and showed up-regulated expression under NH_4_^+^ depletion. VviAMT4;1 is a typical high-affinity ammonium transporter that may recognize NH_4_^+^ but actually transports NH_3_. Both MeA^+^ and L-MSX reduced the growth of yeast mutants transformed with pYES2-*VviAMT4;1*. The VviAMT4;1-mediated ammonium uptake process may be subject to feedback inhibition by endogenous NH_4_^+^ enrichment.

## Data Availability

The data are contained within the present article.

## References

[1] Cruz C., Bio A.F.M., Dominguez-Valdivia M.D., Aparicio-Tejo P.M., Lamsfus C., Martins-Loução M.A. (2006). How does glutamine synthetase activity determine plant tolerance to ammonium?. Planta.

[2] Liu Y., von Wirén N. (2017). Ammonium as a signal for physiological and morphological responses in plants. J. Exp. Bot..

[3] Ludewig U., Neuhäuser B., Dynowski M. (2007). Molecular mechanisms of ammonium transporter and accumulation in plants. FEBS Lett..

[4] McDonald T.R., Ward J.M. (2016). Evolution of electrogenic ammonium transporters (AMTs). Front. Plant Sci..

[5] Straub T., Ludewig U., Neuhäuser B. (2017). The kinase CIPK23 inhibits ammonium transport in *Arabidopsis thaliana*. Plant Cell.

[6] You S.H., Wang Y.Q., Li Y.J., Li Y.H., Tan P., Wu Z., Shi W.J., Song Z.Z. (2020). Cloning and functional determination of ammonium transporter PpeAMT3; 4 in peach. Biomed. Res. Int..

[7] Ninnemann O., Jauniaux J.C., Frommer W.B. (1994). Identification of a high affinity NH_4_^+^ transporter from plants. EMBO J..

[8] Ludewig U., von Wiren N., Frommer W.B. (2002). Uniport of NH_4_^+^ by the root hair plasma membrane ammonium transporter LeAMT1; 1. J. Biol. Chem..

[9] Ludewig U., Wilken S., Wu B.H., Jost W., Obrdlik P., Bakkoury M.E., Marini A., André B., Hamacher T., Boles E. (2019). Homo- and hetero-oligomerization of ammonium transporter-1 NH_4_^+^ uniporters. J. Biol. Chem..

[10] Sonoda Y., Ikeda A., Saiki S., von Wirén N., Yamaya T., Yamaguchi J. (2003). Distinct expression and function of three ammonium transporter genes (*OsAMT1;1-1;3*) in rice. Plant Cell Physiol..

[11] Yang S., Hao D., Cong Y., Jin M., Su Y.H. (2015). The rice OsAMT1; 1 is a proton-independent feedback regulated ammonium transporter. Plant Cell Rep..

[12] Hao D., Yang S., Huang Y., Su Y.H. (2016). Identification of structural elements involved in fine-tuning of the transport activity of the rice ammonium transporter OsAMT1; 3. Plant Physiol. Biochem..

[13] Guo X.T., Sheng Y.T., Yang S.Y., Han L., Gao Y.C., Zhang K., Cheng J.S., Zhang H.X., Su Y.H. (2019). Isolation and characterization of a high-affinity ammonium transporter ApAMT1; 1 in alligatorweed. Plant Growth Regul..

[14] Li T., Liao K., Xu X., Gao Y., Wang Z.Y., Zhu X.F., Jia B.L., Xuan Y.H. (2017). Wheat ammonium transporter (AMT) gene family: Diversity and possible role in host–pathogen interaction with stem rust. Front. Plant Sci..

[15] Yuan L., Loqué D., Kojima S., Rauch S., Ishiyama K., Inoue E., Takahashi H., von Wirén N. (2007). The organization of high-affinity ammonium uptake in *Arabidopsis* roots depends on the spatial arrangement and biochemical properties of AMT1-type transporters. Plant Cell.

[16] Kaiser B.N., Rawat S.R., Siddiqi M.Y., Masle J., Glass A.D.M. (2002). Functional analysis of an *Arabidopsis* T-DNA “knockout” of the high-affinity NH_4_^+^ transporter AtAMT1.1. Plant Physiol..

[17] Loqué D., Yuan L., Kojima S., Gojon A., Wirth J., Gazzarrini S., Ishiyama K., Takahashi H., von Wirén N. (2006). Additive contribution of *AtAMT1.1* and *AtAMT1.3* to high-affinity ammonium uptake across the plasma membrane of nitrogen-deficient *Arabidopsis* roots. Plant J..

[18] Ninkuu V., Liu Z.X., Sun X.W. (2023). Genetic regulation of nitrogen use efficiency in *Gossypium* spp. Plant Cell Environ..

[19] Neuhäuser B., Dynowski M., Ludewig U. (2009). Channel-like NH_3_ flux by ammonium transporter AtAMT2. FEBS Lett..

[20] Hui J., An X., Li Z.B., Neuhäuser B., Ludewig U., Wu X.N., Schulze W.X., Chen F.J., Feng G., Lambers H. (2022). The mycorrhiza-specific ammonium transporter ZmAMT3; 1 mediates mycorrhiza-dependent nitrogen uptake in maize roots. Plant Cell.

[21] Li H., Cong Y., Chang Y.H., Lin J. (2016). Two AMT2-Type ammonium transporters from *Pyrus betulaefolia* demonstrate distinct expression characteristics. Plant Mol. Biol. Rep..

[22] Shi X.B. (2012). Nitrogen Requirement Regularity of *Kyoho* Grapevines and Absorption, Utilization Features of the Different Rootstocks. Master’s Thesis.

[23] Wang J. (2021). The Effect of Exogenous Nitrogen on Wine Grape Physiology and Wine Quality. Master’s Thesis.

[24] Zhang L., Zong Y.Q., Yu W.H., Han L., Sun Y.Z., Chen C.H., Chen S.L., Zhang K., Cheng J.S., Tang M.L. (2021). Identification, cloning, and expression characteristics analysis of Fe-S cluster assembly genes in grape. Sci. Agric. Sin..

[25] Sun X.J., Tao Y.F., Chi L.J., Tang M.L., Guan X.Q., Shi M., Song Z.Z. (2025). Cloning and function determination of cytochrome P450 encoding gene *CYP76F14* from three different flavor-type table grape varieties. Sino-Overseas Grapevine Wine.

[26] Tang M.L., Li Y.H., Chen Y.H., Han L., Zhang H.X., Song Z.Z. (2020). Characterization and expression of ammonium transporter in peach (*Prunus persica*) and regulation analysis in response to external ammonium supply. Phyton-Int. J. Exp. Bot..

[27] Yang Y., Zhang J.J., Li M.Y., Ning Y.Z., Tao Y.F., Shi S.P., Dark A., Song Z.Z. (2023). Heterologous expression of a Ferritin homologue gene *PpFer1* from *Prunus persica* enhances plant tolerance to iron toxicity and H_2_O_2_ stress in *Arabidopsis thaliana*. Plants.

[28] Xia G., Shi M., Xu W., Dark A., Song Z. (2024). Cytochrome P450 VvCYP76F14 dominates the production of wine bouquet precursors in wine grapes. Front. Plant Sci..

[29] Peng B., Ran J.G., Li Y.Y., Tang M.L., Xiao H.L., Shi S.P., Ning Y.Z., Dark A., Guan X.Q., Song Z.Z. (2024). Site-directed mutagenesis of VvCYP76F14 (cytochrome P450) unveils its potential for selection in wine grape varieties linked to the development of wine bouquet. J. Agric. Food Chem..

[30] Song Z.Z., Tang M.L., Xiao H.L., Xu H.H., Shi M., Dark A., Xie Z.Q., Peng B. (2025). Unraveling the trisubstrate-triproduct reaction mechanisms of wine grape VvCYP76F14 to improve wine bouquet. Food Chem..

[31] Selle A., Willmann M., Grunze N., Gessler A., Weiss M., Nehls U. (2005). The high-affinity polar ammonium importer PttAMT1.2 and its role in ectomycorrhizal symbiosis. New Phytol..

[32] Couturier J., Montanini B., Martin F., Brun A., Blaudez D., Chalot M. (2017). The expanded family of ammonium transporters in the perennial poplar plant. New Phytol..

[33] Lima J.E., Kojima S., Takahashi H., von Wirén N. (2010). Ammonium triggers lateral root branching in *Arabidopsis* in an AMMONIUM TRANSPORTER1;3-dependent manner. Plant Cell.

